# ﻿A new species of the genus *Scorpiops* Peters, 1861, subgenus *Euscorpiops* Vachon, 1980 from Thailand (Scorpiones, Scorpiopidae)

**DOI:** 10.3897/zookeys.1193.113398

**Published:** 2024-03-06

**Authors:** Wasin Nawanetiwong, Ondřej Košulič, Natapot Warrit, Wilson R. Lourenço, Eric Ythier

**Affiliations:** 1 Department of Biology, Faculty of Science, Chulalongkorn University, Bangkok, 103303, Thailand Chulalongkorn University Bangkok Thailand; 2 Department of Forest Protection and Wildlife Management, Faculty of Forestry and Wood Technology, Mendel University in Brno, Zemědělská 3, Brno, Czech Republic Mendel University in Brno Brno Czech Republic; 3 Muséum national d’Histoire naturelle, Sorbonne Universités, Institut de Systématique, Evolution, Biodiversité (ISYEB), UMR7205-CNRS, MNHN, UPMC, EPHE, CP 53, 57 rue Cuvier, 75005 Paris, France Sorbonne Universités Paris France; 4 BYG Taxa, 382 rue des Guillates, 71570 Romanèche-Thorins, France BYG Taxa Romanèche-Thorins France

**Keywords:** Description, Kaeng Krachan National Park, morphology, scorpion, Southeast Asia, wet forest

## Abstract

A new species, Scorpiops (Euscorpiops) krachan**sp. nov.**, belonging to the family Scorpiopidae Kraepelin, 1905 is described based on three adult males and one adult female collected in the Kaeng Krachan National Park, Phetchaburi Province, Thailand. The new species presents most features exhibited by scorpions of the subgenus Euscorpiops and can be characterized notably by a very small size, a sexual dimorphism strongly marked with male pedipalps elongated, a distinct trichobothrial pattern and other morphological features. This new taxon may represent one endemic element for the scorpion fauna of Thailand. Aspects of the ecology and distribution of the new species are discussed and compared with that of other relative *Scorpiops* species.

## ﻿Introduction

As already discussed in several previous papers (e.g. Lourenço and Košulič 2018; Lourenço 2019; Lourenço and Ythier 2022), the generic composition of the now accepted family Scorpiopidae was mainly due to Vachon (1980), who revised the genus *Scorpiops* Peters, 1861 and described three new subgenera (*Alloscorpiops*, *Euscorpiops*, and *Neoscorpiops*) in addition to the nominotypical subgenus Scorpiops. These four subgenera were later elevated to generic rank by Lourenço (1998), and new taxa of the generic level were subsequently added to the family (*Parascorpiops* Banks, 1928, *Dasyscorpiops* Vachon, 1974, *Laoscorpiops* Lourenço, 2013, *Vietscorpiops* Lourenço & Pham, 2015, and *Plethoscorpiops* Lourenço, 2017). Later, Kovařík (2000) rejected the validity of the genus *Euscorpiops*, which was subsequently reestablished by Soleglad and Sissom (2001), mainly based on the position of chelal trichobothrium *Eb3* and the presence of an annular ring on the telson. The generic composition of the family Scorpiopidae was then globally well accepted for about 20 years until Kovařík et al. (2020) simply decided to place all the known and accepted genera of the family in the synonymy of *Scorpiops*, with the single exception of *Parascorpiops*. This drastic decision was not accepted by Lourenço and Ythier (2022), who restored some division of the family Scorpiopidae by revalidating, as subgenera of the genus *Scorpiops*, several previously defined genera: *Alloscorpiops*, *Euscorpiops*, *Neoscorpiops*, *Dasyscorpiops*, and *Plethoscorpiops*. In the same paper, *Parascorpiops* was maintained as a distinct genus, while the synonymy of *Laoscorpiops* and *Vietscorpiops* with *Alloscorpiops* and *Scorpiops*, respectively, was maintained. In the present study, a new species belonging to the subgenus Euscorpiops is described from Kaeng Krachan National Park located in Phetchaburi Province of Central Thailand. This new taxon may represent an endemic element of the scorpion fauna of Thailand.

## ﻿Methods

Illustrations and measurements were made using a Wild M5 stereomicroscope with a drawing tube and an ocular micrometer, a Canon EOS 7D camera, and Adobe Photoshop software. The map was made using QGIS and Adobe Photoshop. Measurements follow Stahnke (1970) and are given in millimeters. Trichobothrial notations follow Vachon (1974) and morphological terminology mostly follows Vachon (1952) and Hjelle (1990). A collecting permit was provided by the Department of National Parks, Wildlife and Plant Conservation, Ministry of Natural Resources and Environment in Thailand.

## ﻿Taxonomic treatment

**Family Scorpiopidae Kraepelin, 1905**

**Genus *Scorpiops* Peters, 1861**

**Subgenus Euscorpiops Vachon, 1980**

### ﻿ Scorpiops (Euscorpiops) krachan

Taxon classificationAnimaliaScorpionesScorpiopidae

Nawanetiwong, Košulič, Warrit, Lourenço & Ythier sp. nov.

1582763A-3DFB-57E1-89DE-A9C1C154D5FA

https://zoobank.org/53269528-C6AC-44AE-AB99-FF60D486DF8E

Figs 1 , 2 , 3

#### Type locality.

Thailand, Phetchaburi Province: Kaeng Krachan National Park, Ban Krang Campsite, 12°47.970'N, 99°27.236'E, 324 m a.s.l., wet secondary forest (cloud-forest), 14 Nov. 2022, O. Košulič leg.

**Figure 1.**
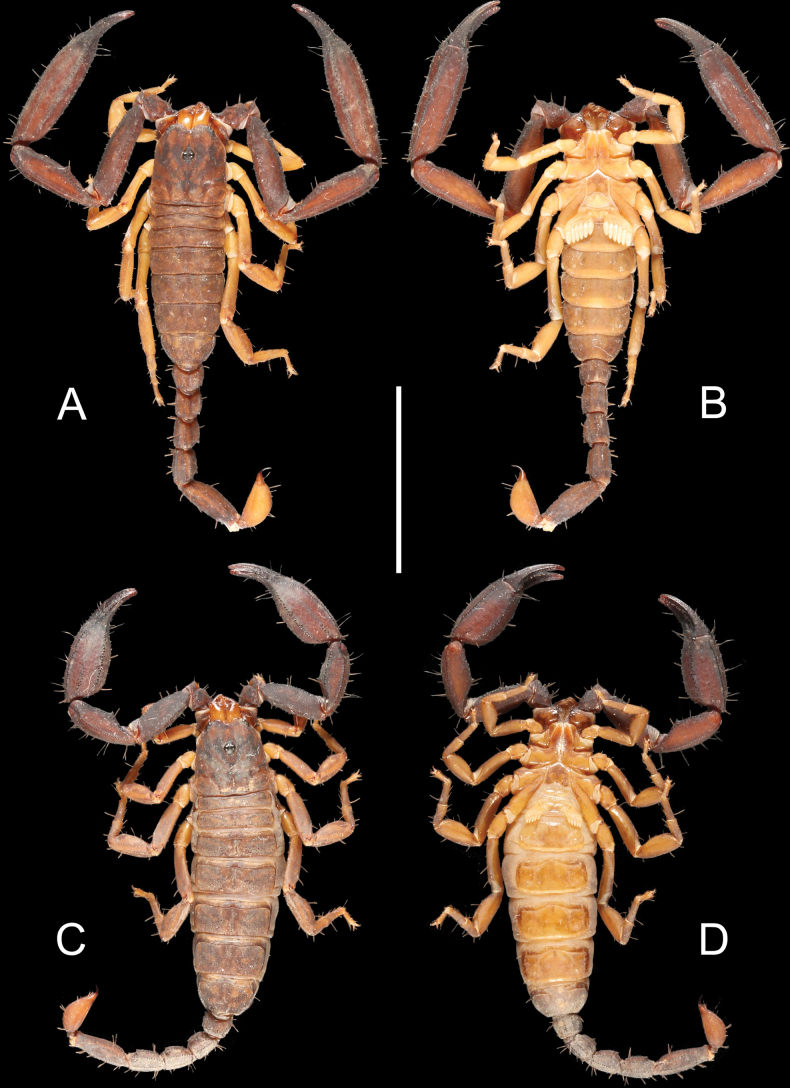
Scorpiops (Euscorpiops) krachan sp. nov. **A, B** male holotype, habitus, dorsal **A** and ventral **B** aspects **C, D** female paratype, habitus, dorsal **C** and ventral **D** aspects. Scale bar 1 cm.

#### Type specimen.

***Holotype***, ♂. Original label: almost same as designation in type locality, deposited at the Muséum national d’Histoire naturelle, Paris, France. • ***Paratypes***: 1 ♀. same data as holotype; 2 ♂. Original label: Thailand: Phetchaburi Province, Kaeng Krachan National Park, Ban Krang Campsite, 12°47.948'N, 99°27.250'E, 317 m a.s.l., wet secondary forest (cloud-forest), 14 Nov. 2022, O. Košulič leg., deposited at Department of Biology, Faculty of Science, Chulalongkorn University, Bangkok, Thailand (SCO-2022-005, SCO-2022-006).

#### Etymology.

The specific name refers to the National Park of Kaeng Krachan where the new species was collected.

#### Diagnosis.

The new species exhibits the general characteristics of the subgenus Euscorpiops (Vachon 1980; Soleglad and Sissom 2001). Total length of male and female 21.7–26.9 and 25.9 mm, respectively, defining the new species as very small in comparison to most other species of the subgenus. General coloration brownish yellow; female darker than male; chelicerae yellow without any variegated spots. Pectines with 6–7 and 5–5 teeth in male and female, respectively; two marginal and two middle lamellae present; fulcra present. Sexual dimorphism strongly marked, with male pedipalps markedly elongated; chela length/width ratio 4.5–5.1 in male, 3.0 in female. Chelal fingers straight in both sexes; movable fingers with two parallel longitudinal rows of granules almost fused, formed by a row of about 50 median granules and a row of 19–20 inner granules (4–5) and inner accessory granules (15); 7–8 outer granules are present. Annular ring conspicuous in both sexes; telson length/depth ratio 2.6–2.8 in male, 2.6 in female. Trichobothriotaxy of type C (Vachon 1974, 1980); three trichobothria on femur (dorsal, internal, and external); patella with two dorsal, one internal, six ventral, and 16(15) external trichobothria; chelal manus with four ventral, two dorsal (*Dt*, *Db*), two internal (*ib*, *it*), one *Est*, five *Et*, one *Esb*, and three trichobothria in the *Eb* series; trichobothrium *Eb_3_* located in distal half of manus, between trichobothria *Dt* and *Est*.

#### Description.

Based on male holotype and female and male paratypes.

#### Coloration.

Basically yellowish to brownish yellow. Carapace brownish yellow, with paler zones posteriorly and on furrows. Tergites brownish yellow. Metasomal segments brownish yellow, darker in female; telson yellow; base of aculeus blackish and tip reddish. Chelicerae yellow, without any variegated spots; one blackish spot at the base of fixed finger; fingers brownish yellow, with reddish teeth. Pedipalps reddish brown to brownish, darker in female; fingers darker than chela manus, almost blackish. Legs yellow, intensely spotted with brownish. Venter yellow; coxapophysis, sternum and sternites markedly infuscated.

#### Morphology.

**Figure 2.**
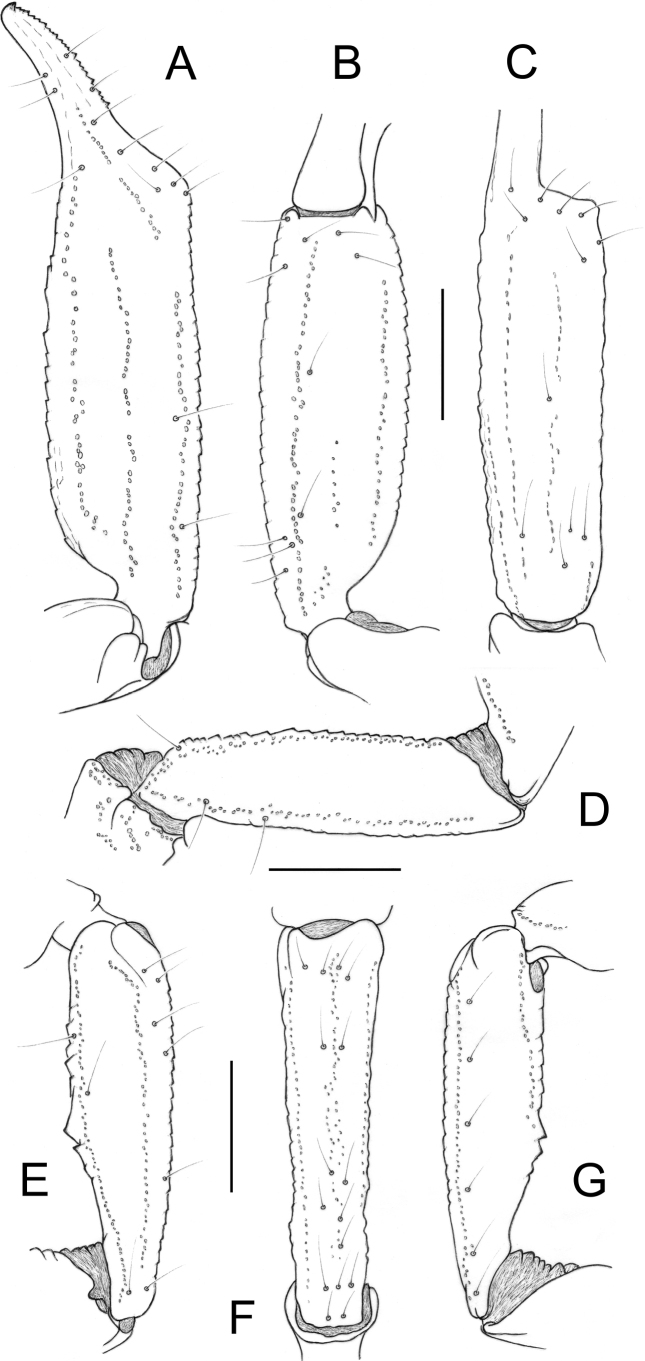
Scorpiops (Euscorpiops) krachan sp. nov. Male holotype, trichobotrial pattern **A–C** chela, dorso-external **A** ventral **B** and external **C** aspects **D** femur, dorsal aspect **E–G** patella, dorsal **E** external **F** and ventral **G** aspects. Scale bars 2 mm.

Carapace weakly granular, rather shagreened; furrows weakly deep. Median eyes anterior to the middle of carapace; three pairs of lateral eyes, the posterior one small in female and relictual in male. Sternum pentagonal, slightly longer than wide. Tergites weakly granulated, mostly shagreened; VII with five carinae, moderately marked; median carinae vestigial. Pectines large in male and reduced in female with a pectinal tooth count of 6-6 and 5-5, respectively; two marginal and two middle lamellae present; fulcra present. Sternites almost smooth and slightly punctated, with round spiracles; sternite VII with four vestigial carinae and some granulations, better marked in male. Metasomal segments I to V with 10-8-8-8-7 carinae; dorsal carinae on segments II–IV with several spinoid granules and one larger posterior spinoid granule; metasomal tegument weakly granulated; ventral carina on segment V with weakly marked spinoid granules. Telson vesicle with minute granulations, but largely smooth; annular ring conspicuous; telson length/depth ratio 2.6–2.8 in male, 2.6 in female. Setation moderate on metasomal segments and telson. Pedipalps: femur with dorsal internal, dorsal external, ventral internal and ventral external carinae moderately marked; tegument weakly granular. Patella with dorsal internal, dorsal external, ventral internal, ventral external, and external carinae weakly marked; one moderately to weakly marked spinoid granule present on internal aspect; tegument weakly granular. Chela with dorsal marginal, external secondary, ventral internal, and ventral carinae moderately marked; other carinae weak; tegument weakly granulated. Sexual dimorphism strongly marked with male pedipalps markedly elongated; chela length/width ratio 4.5–5.1 in male, 3.0 in female. Chelal fingers straight in both sexes; movable fingers with two parallel longitudinal rows of granules almost fused, formed by a row of about 50 median granules and a row of 19–20 inner granules (4–5) and inner accessory granules (15); 7–8 outer granules present. Cheliceral dentition as defined for the family (Vachon 1963); a few teeth on ventro-internal face of movable finger. Trichobothriotaxy of type C, as shown in Fig. 2 (Vachon 1974, 1980); three trichobothria on femur (dorsal, internal, and external); patella with two dorsal, one internal, six ventral, and 16(15) external trichobothria; chelal manus with four ventral, two dorsal (*Dt*, *Db*), two internal (*ib*, *it*), one *Est*, five *Et*, one *Esb*, and three trichobothria in the *Eb* series. Trichobothrium *Eb_3_* distal in relation to *Eb_2_* (Vachon 1974, 1980), located in distal half of manus, between trichobothria *Dt* and *Est*. Legs tarsi with 4–5 long setae; tibial spurs absent.

#### Morphometric values.

Male holotype and female paratype of Scorpiops (Euscorpiops) krachan sp. nov. Total length including the telson 26.9/25.9. Carapace: length 4.2/4.1; anterior width 2.7/2.5; posterior width 4.2/4.2. Mesosoma length 8.6/9.8. Metasomal segments. I: length 1.3/1.2, width 1.7/1.5; II: length 1.6/1.4, width 1.5/1.3; III: length 1.8/1.6, width 1.4/1.2; IV: length 2.2/2.0, width 1.3/1.1; V: length, 3.6/2.9, width 1.1/1.0, depth 1.2/1.1. Telson length 3.6/2.9; vesicle: width 1.6/1.2, depth 1.3/1.1. Pedipalp: femur length 5.8/3.9, width 1.4/1.4; patella length 6.1/4.2, width 1.5/1.4; chela length 9.5/6.9, width 2.1/2.3, depth 1.8/1.9. Movable finger length 3.1/2.9.

**Figure 3.**
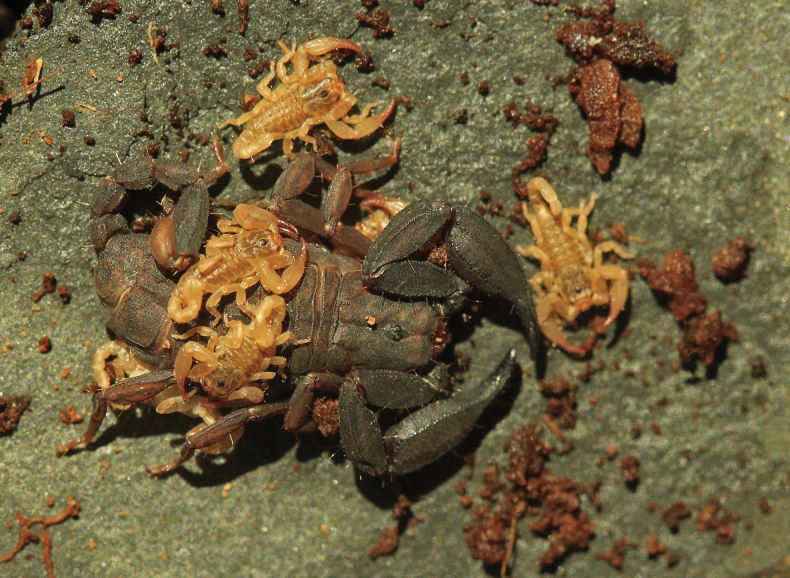
Scorpiops (Euscorpiops) krachan sp. nov., alive with pre-juveniles (instar I).

#### Relationships.

**Figure 4.**
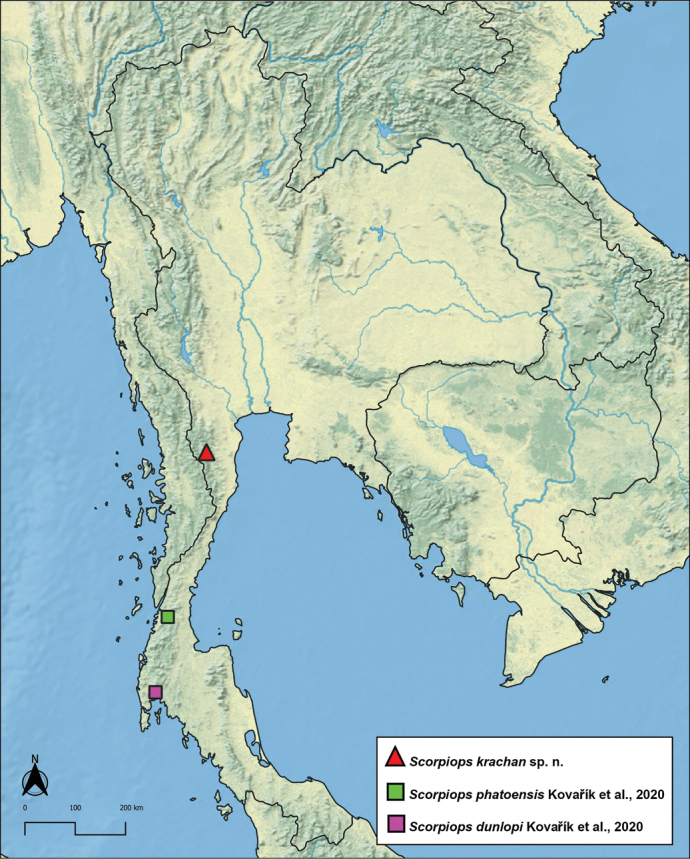
Distribution map showing the type localities of the new species and its most relative *Scorpiops* species: S. (Euscorpiops) krachan sp. nov. (red triangle), *S.phatoensis*Kovařík et al., 2020 (green square), *S.dunlopi*Kovařík et al., 2020 (purple square).

The most similar species seem to be Scorpiops (Euscorpiops) phatoensis and Scorpiops (Euscorpiops) dunlopi, both described by Kovařík et al. (2020) from South Thailand (Fig. 4), notably based on size, number of pectine teeth, marginal and middle lamellae, shape of fingers, and number of external trichobothria on patella. *Scorpiopskrachan* sp. nov. can, however, be separated from these two species by the following main features:

1. lighter coloration pattern (reddish brown to reddish black in *S.phatoensis* and *S.dunlopi*);
2. chelicerae without any variegated spots (variegated in *S.phatoensis* and *S.dunlopi*);
3. pectines with fulcra present (absent in *S.phatoensis* and reduced in *S.dunlopi*);
4. male chela slenderer than in *S.phatoensis* with length to width ratio 4.5–5.1 (3.7 in *S.phatoensis*);
5. chelal movable fingers with about 50 median granules (about 40 in *S.phatoensis* and 35 in *S.dunlopi*), 4–5 inner granules (5–7 in *S.phatoensis* and absent in *S.dunlopi*), 15 inner accessory granules (about 10 in *S.phatoensis* and 10–12 in *S.dunlopi*) and 7–8 outer granules (absent in *S.dunlopi*);
6. female telson less elongated with length to depth ratio 2.6 in female (2.8–3.0 in *S.phatoensis* and 3.1 in *S.dunlopi*);
7. chelal manus trichobothrium *Eb _3_* located in distal half of manus, between trichobothria *Dt* and *Est* (located in middle of manus, at same level or distal to *Dt* in *S.phatoensis*);
8. an allopatric geographic distribution (type localities of *S.phatoensis* and *S.dunlopi* about 350 km and 500 km to the south, respectively).

Another species, Scorpiops (Euscorpiops) binghamii Pocock, 1893, described from southern Myanmar, is geographically close to *S.krachan* sp. nov. but can easily be distinguished from the new species, notably by the number of external trichobothria on patella (20–21), whereas *S.krachan* sp. nov. has 15–16.

## ﻿Distribution and ecological affinities of *Scorpiops* species in Thailand

The members of this genus can be found in altitudes ranging from 40 to 1800 m a.s.l. (Kovařík 1993, 2000, 2013; Kovařík et al. 2013a, 2013b, 2015, 2020; Lourenço 2019). Habitats are mostly covered with various forest types from deciduous forest to lower mountain forest (Maxwell 2004). *Scorpiopskrachan* sp. nov. is the second species reported from Phetchaburi province; the other species is *S.anthracinus* (Kovařík et al. 2020). *Scorpiopskrachan* sp. nov. inhabits the Tenasserim Mountain Range, which is covered with rainforest (wet forest including secondary and primary forests), similar to other *Scorpiops* species found in the country.

**Figure 5.**
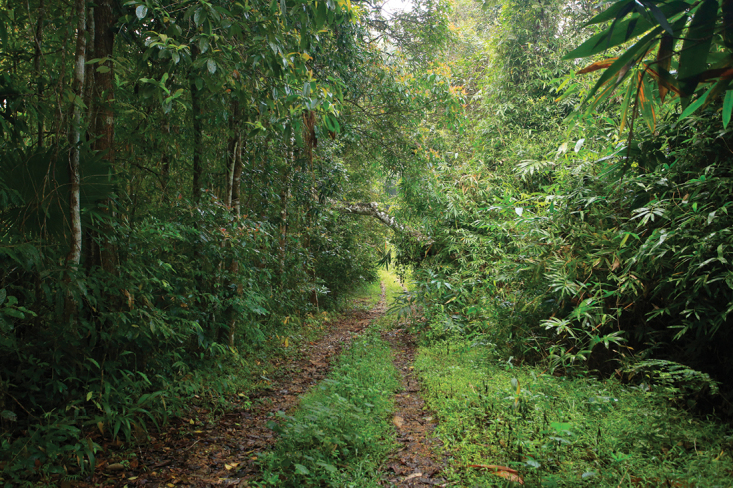
Natural habitat of Scorpiops (Euscorpiops) krachan sp. nov. in Kaeng Krachan National Park, Phetchaburi Province, Thailand.

The microhabitats of *Scorpiops* species in Thailand include soil walls, dead logs, piled stones, and buffalo feces (Kovařík 1993). These microhabitats can often be found near caves. *Scorpiopskrachan* sp. nov. was collected underneath a rock in transitional habitat between secondary to primary forests (Fig. 5). In the same habitat, several subadult specimens of *Heterometrus* scorpions, most likely belonging to *H.minotaurus* Plíšková, Kovařík, Košulič & Šťáhlavský, 2016, were found.

*Scorpiops* microhabitats suggest that these predators have an ambush or sit-and-wait type of foraging (McCormick and Polis 1990). It is predicted that scorpions in this genus have limited distribution ranges with high degree of endemism, as it has been reported for *S.dunlopi*, *S.phatoensis* (Kovařík et al. 2020), and S. (Alloscorpiops) viktoriae (Lourenço and Košulič 2018).

Until now, all *Scorpiops* species reported in Thailand were believed to be endemic to their habitats (Kovařík 1993, 2000, 2013; Kovařík et al. 2013a, 2013b, 2015, 2020; Lourenço 2019). They can be found mainly in mountain areas, particularly in places with numerous rock crevices. Despite several studies on *Scorpiops* in Thailand, additional areas of the country are still unexplored and needed investigation. We suggest that future studies focus on cavernous mountainous habitats.

## Supplementary Material

XML Treatment for Scorpiops (Euscorpiops) krachan

## ﻿Additional information

### Author ORCIDs

Ondřej Košulič https://orcid.org/0000-0003-2199-1694

Natapot Warrit https://orcid.org/0000-0002-6338-1782

Wilson R. Lourenço https://orcid.org/0000-0002-2386-363X

Eric Ythier https://orcid.org/0000-0002-3194-5184

### Data availability

All of the data that support the findings of this study are available in the main text.
